# A Novel Scoring System for Risk Assessment of Elderly Patients With Cytogenetically Normal Acute Myeloid Leukemia Based on Expression of Three AQP1 DNA Methylation-Associated Genes

**DOI:** 10.3389/fonc.2020.00566

**Published:** 2020-04-21

**Authors:** Xuejiao Yin, Haifan Huang, Sui Huang, Aoshuang Xu, Fengjuan Fan, Shanshan Luo, Han Yan, Lei Chen, Chunyan Sun, Yu Hu

**Affiliations:** ^1^Institute of Hematology, Union Hospital, Tongji Medical College, Huazhong University of Science and Technology, Wuhan, China; ^2^Collaborative Innovation Center of Hematology, Huazhong University of Science and Technology, Wuhan, China

**Keywords:** elderly, CN-AML, AQP1, methylation, gene regulation, lncRNA, prognosis

## Abstract

**Background:** Aquaporin 1 (AQP-1), a transmembrane water channel protein, has been proven to involve in many diseases' progression and prognosis. This research aims to explore the prognostic value of AQP-1 in elderly cytogenetically normal acute myeloid leukemia (CN-AML).

**Methods:** Complete clinical and expression data of 226 elderly patients (aged > 60) with cytogenetically normal acute myeloid leukemia (CN-AML) were downloaded from the databases of The Cancer Genome Atlas (TCGA) and Gene Expression Omnibus (GEO). We have explored prognostic significance of AQP-1, investigated the underlying mechanism, and developed a novel scoring system for the risk assessment of elderly patients with AML based on AQP1 methylation.

**Results:** In the first and second independent group, AQP1 shows lower expression in CN-AML than normal people, while high AQP1 expression and AQP1 promoter hypomethylation were related to better overall survival (OS; *P* < 0.05). To understand the underlying mechanisms, we investigated differentially expressed genes (DEGs), miRNA and lncRNA associated with AQP1 methylation. A three-gene prognostic signature based on AQP1 methylation which was highly correlated with OS was established, and the performance was validated by Permutation Test and Leave-one-out Cross Validation method. Furthermore, an independent cohort was used to verify the prognostic value of this model.

**Conclusions:** AQP1 methylation could serve as an independent prognostic biomarker in elderly CN-AML, and may provide new insights for the diagnosis and treatment for elderly CN-AML patients.

## Introduction

Acute myeloid leukemia (AML) is a heterogeneous hematologic malignancy characterized by invasion of the bone marrow, blood, and other tissues by myeloid progenitor cells with enhanced proliferative capabilities. AML is more commonly diagnosed in the elderly with a median age of around 67, and approximately one third of the patients are 75 or older (1). The Surveillance, Epidemiology, and End Results (SEER) Program of the National Cancer Institute reported that in the United States, the incidence of AML is 4.3 per 100,000 persons, while it increases to 12.2 per 100,000 among those aged 65–69 and 28.5 per 100,000 for those aged 80–84 (2). The current National Comprehensive Cancer Network (NCCN) Guidelines for AML recommend intensive induction therapy, followed by consolidation and a possible allogeneic stem cell transplantation (alloSCT) after which 85% of the patients aged between 18 and 60 could achieve complete remissions (CR) (3, 4). Due to advanced age, comorbidities, preexisting myelodysplasia and poor performance status, the elderly patients are often intolerable to standard treatments and more likely to undertake less intensive therapies or supportive care, which often lead to inferior outcomes (lower CR rate, shorter remissions and OS) (5). The most common type of AML is the cytogenetically normal acute myeloid leukemia (CN-AML), a group without microscopically discernible chromosome aberrance, nevertheless, there could still be some genetic mutations, dysregulated expression and epigenetic alterations (6). Recurrent mutated genes in elderly CN-AML patients include NPM1, CEBPA, FLT3-ITD, and WT1, which are associated with different prognostic significance. Advances in high-throughput technologies, such as microarray and next-generation sequencing (NGS), have expanded our understanding of the roles of genetic markers in AML, and were used in refining risk stratification and treatment selection in young and middle-aged patients (7). However, less such progress was seen in elderly AML. High-throughput technologies could help to identify underlying molecular mechanisms associated with progression and prognosis of the disease, which might lead to developments in targeted treatment and improve patients' outcomes.

DNA methylation, an important regulator of gene expression, is the most studied epigenetic modification. DNA methylation plays critical roles in diverse biological functions of cancer progress including disease initiation, promotion, invasion, metastases, and chemotherapy resistance (8). Abnormal DNA methylation has been found to be a hallmark of AML (9), and a suitable biomarker to predict prognosis (10).

The aquaporin 1 (AQP-1) gene, located on chromosome 7p14, encodes a highly conserved transmembrane water channel protein with a molecular weight of 28 kDa, and facilitates transcellular water transportation (11). AQP1 plays an oncogenic role in many types of solid cancer, including colorectal cancer, breast cancer, bladder cancer (12–14). Functionally, AQP1 regulates cell proliferation, invasion, metastasis and angiogenesis. AQP1 is strongly associated with many important tumor signaling pathways that promote cell proliferation and contribute to carcinogenesis, such as NF-κB (15, 16), Notch (17), PI3K/Akt (18), and p38-MAPK pathways (19). Furthermore, hypomethylation of AQP1 promoter was common in adenoid cystic carcinoma, and was a newly found biomarker related with prognosis and recurrence of the disease (20). However, one recent study shows that AQP1 acts as a tumor suppressor gene and down-regulate Wnt signaling by interacting with b-catenin, GSK3b, LRP6, and Axin1 (21).

To date, the methylation pattern, expression and clinical significance of AQP1 in elderly CN-AML patients haven't been explored. Therefore, here we explored prognostic significance and mechanisms of AQP1, as well as AQP1 methylation-associated genes in elderly CN-AML patients. The study aimed to improve the understanding of AQP1 in the pathogenesis of elderly AML, and provide potential diagnostic biomarkers for clinical treatment.

## Methods

### Patients and Datasets

43 elderly CN-AML patients (age > 60) only with RNA sequencing data (IlluminaHiSeq_RNASeqV2), 39 elderly CN-AML patients (age > 60) only with DNA methylation data (Illumina Human Methylation 450 K) profiles, 29 elderly CN-AML patients (age > 60) with DNA methylation data (Illumina Human Methylation 450 K) profiles and RNA sequencing including lncRNA and mRNA as well as 20 elderly CN-AML patients (age > 60) with DNA methylation data (Illumina Human Methylation 450 K) profiles and miRNA expression (IlluminaHiSeq_miRNASeq) were gathered from the database of The Cancer Genome Atlas (TCGA).

Five different GEP data sets from the Gene Expression Omnibus (GEO) database were included: (1) GSE1159 including 5 healthy donors and 116 newly diagnosed CN-AML patients; (2) GSE16432 including 34 elderly CN-AML patients(age > 60); (3) GSE23312 including 28 elderly CN-AML patients(age > 60); (4) GSE16432 including 31 patients with *t*_(8,21)_; (5) GSE23312 including 34 patients with *t*_(15,17)_; (6) GSE3224 including 47 patients with inv16; (7) GSE16432 including 13 patients with +8; (8) GSE22778 including 44 patients with complex karyotype; (9) GSE22778 including 148 younger CN-AML patients(age < 60); (10) GSE22778 including 42 elderly CN-AML patients(age > 60). The study was in complete compliance with the publication instructions from TCGA and GEO. Because the data was collected from GEO and TCGA, there's no need for the approval of ethics committees.

149 young patients with AML (age < 30) were collected from target database.

### Data Processing

Samples were divided into AQP1 hypermethylation and AQP1 hypomethylation groups according to the median AQP1 methylated value of 8.667. The differentially expressed lncRNA, mRNA and miRNA between the two groups were identified by the edgeR package in R Bioconductor. The criteria in significant differences was: |log_2_ fold change (FC)|>2 and adjusted *P*-value (padj) ≤0.05. Next, DEGs based on AQP1 methylation were put into univariate cox's model and *P* < 0.05 was set as the significance threshold. Univariate cox analysis was also applied to test clinical information with the same cutoffs, including gender, age at diagnosis, FAB classifications, molecular mutations (NPMc, FLT3-ITD, IDH1), peripheral blasts and bone marrow blasts. The relationship between differentially methylated sites and the expression of AQP1 was computed via pairwise Pearson correlation coefficients, and *p* < 0.05 with *r* < −0.3 was considered as significantly correlated methylation site-gene pairs.

### Signature Development

The risk score was computed on the basis of each gene's expression and their contribution on overall survival denoted by the coefficient of β in a Cox multivariate model. The risk score = β1G1 + β2G2 + β3G3+ ……βnGn (G: each gene's expression value). Next, patients were divided into high risk or low risk group on the basis of median calculated scores. Kaplan-Meier method was carried out to compare survival time between high risk and low risk group with *P* < 0.05. Heat map and ROC curve were applied to assess the prognostic efficacy of the model.

### Permutation Test and Leave-One-Out Cross Validation (LOO-CV)

#### Permutation Test

The label of each patient's characteristics in our study included survival status, overall survival time and a risk score computed via 3-gene prognostic signature. With the risk score in line with each individual, a stochastic system was established by assigning labels to individuals randomly. The stochastic system was examined for survival significance, and it failed to predict the prognosis of patients if the model worked well. The area under Receiver Operating Characteristic curve (AUC) was assumed to be equal to 0.5. A thousand stochastic systems were created via R Bioconductor. After all iterance, we consider *P*-value with a cutoff at 0.05 as a criterion to evaluate the significance between AUC of stochastic systems and the right label system. The 3-gene signature was thought to have no effects on the outcome if the *P*-value calculated was >0.05.

#### LOO-CV

Briefly, one observation was precluded each time and the rest was applied to construct a model with 3 genes described above, while a prediction was made for the excluded one. We have carried out 29 tests and the average AUC.

### GO, Pathway Analysis, and PPI Network Establishment

Gene Ontology (GO) and Kyoto Encyclopedia of Genes and Genomes (KEGG) pathway analysis were conducted via the Database for Annotation, Visualization and Integrated Discovery (DAVID, http://david.abcc.ncifcrf.gov/), which provides functional interpretation of various genes originated from genomic researches. Protein–protein interaction (PPI) network of DEGs was set up via Search Tool for the Retrieval of Interacting Genes/Proteins (STRING, http://string.embl.de/), which offers systemic perspective of cellular processes. “Co-expression value ≥0.7” was considered as the cut-off point.

## Results

### Identification of Methylation Dependent AQP1 Gene Related With Prognosis of Elderly CN-AML

**Figure 1:**
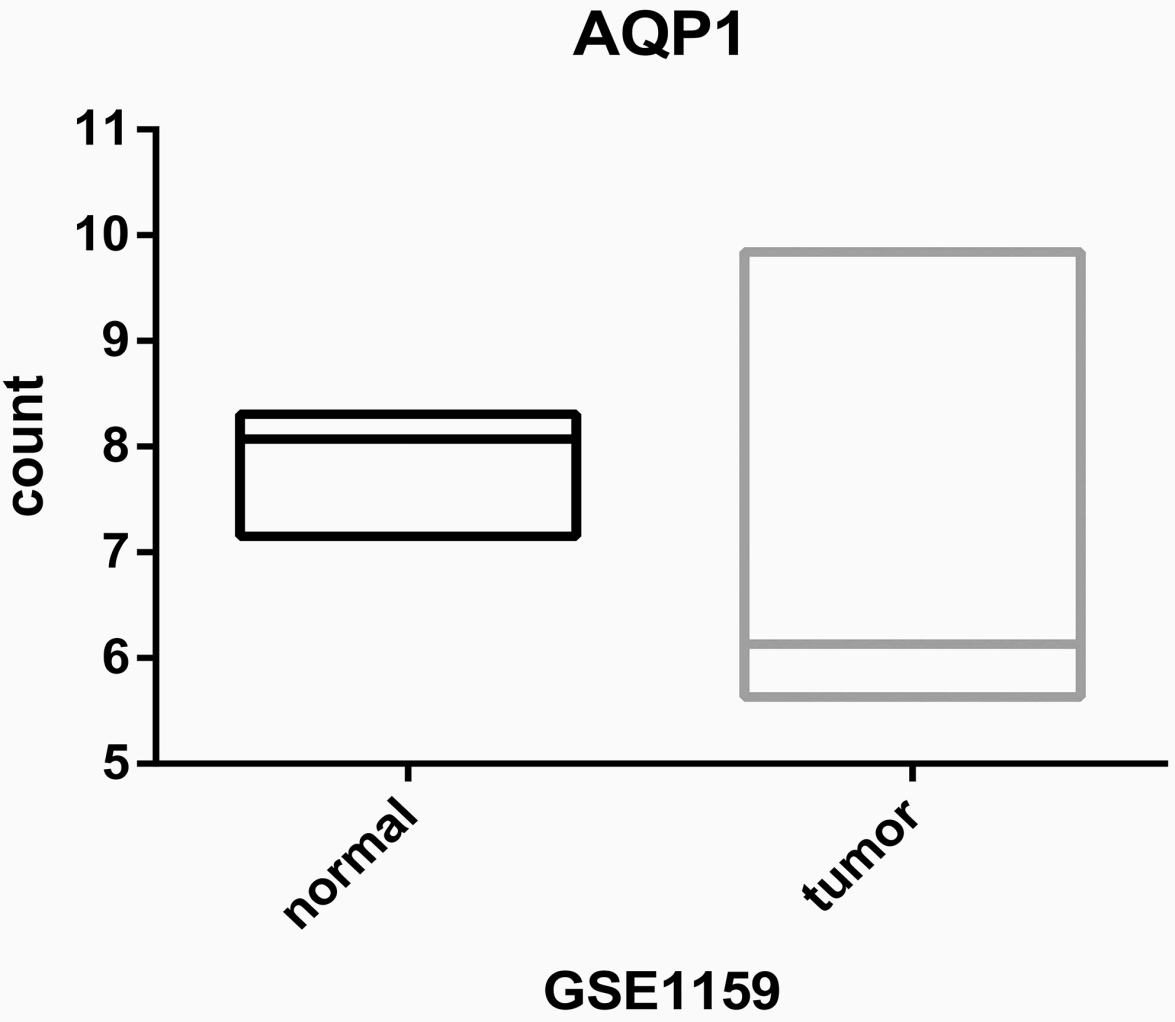
Differential expression of AQP1 between CN-AML-BM cases (*n* = 116) and NBM samples (*n* = 5).

**Figure 2:**
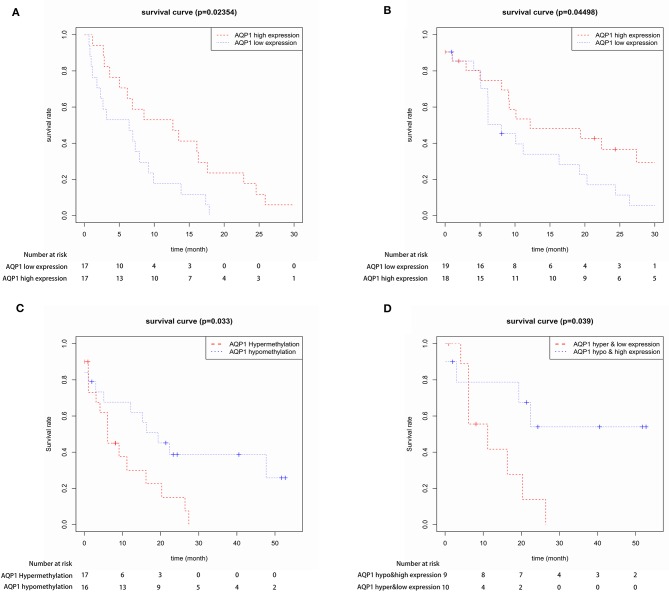
The prognostic value of AQP1 expression in 34 elderly CN-AML patients from GSE16432 **(A)** and in 43 elderly CN-AML patients from TCGA **(B)**. The prognostic value of AQP1 methylation in 39 elderly CN-AML patients with DNA methylation data from TCGA **(C)** and in 29 elderly CN-AML patients with DNA methylation data and RNA sequencing data from TCGA **(D)**.

First, we evaluated the AQP1 expression in elderly CN-AML. AQP1 expression was significantly downregulated in BM from the primary cohort of 116 CN-AML patients compared with normal bone marrow (BM) (*p* < 0.05, Figure 1, 116 CN-AML vs. 5 normal BM, GEO No: GSE1159). Moreover, we found that there was no significant difference in the expression level of AQP1 between younger and elderly CN-AML patients (Figure S1, GEO No: GSE22778). To investigate the prognostic value of AQP1 in elderly CN-AML (age > 60), we divided the 34 elderly CN-AML patients (age > 60) from GSE16432 into 2 groups according to the median level of AQP1 expression. The results demonstrated that high AQP1 expression group had significantly longer overall survival (OS) (*P* = 0.02354, Figure 2A, 34 elderly CN-AML, GEO No: GSE16432). The association between AQP1 expression and prognostic significance was further validated by another microarray dataset of TCGA (*P* = 0.04498, Figure 2B, 43 elderly CN-AML with RNA sequencing, TCGA).

We further assessed and confirmed the direct correlation between AQP1 methylation and expression in elderly CN-AML from TCGA database (Figure S2). Next, we analyzed the prognostic significance of AQP1 methylation in elderly CN-AML from TCGA database. AQP1 hypomethylation group had longer OS than AQP1 hypermethylation group (*P* = 0.033, Figure 2C, 39 elderly CN-AML with DNA methylation data, TCGA), and AQP1 hypomethylation and high expression was further verified to have prognostic significance (*P* = 0.039, Figure 2D, 29 elderly CN-AML with DNA methylation data and RNA sequencing, TCGA). These data suggested that AQP1 hypomethylation was a potential valid prognostic marker.

In order to further analyze the association between AQP1 methylation and clinical features in elderly CN-AML, we compared the clinical and laboratory features between AQP1 hypermethylated and AQP1 hypomethylated groups using 8.667 as the median AQP1 methylated value (Figure S3, 39 elderly CN-AML with DNA methylation data, TCGA). AQP1 hypermethylation was correlated with higher peripheral blasts. However, there was no significant association between AQP1 methylation status and gender, WBC at diagnosis, bone marrow blasts, age at diagnosis, FAB classifications or platelets counts. In addition, no links were found between AQP1 methylation level and FLT3 or IDH1 mutations, while AQP1 hypermethylation was more prevalent in patients carrying NPMc mutations compared to non-carriers (Figure S3).

### Identification and Validation of a Three-Gene Prognostic Signature in Two Datasets

**Figure 3:**
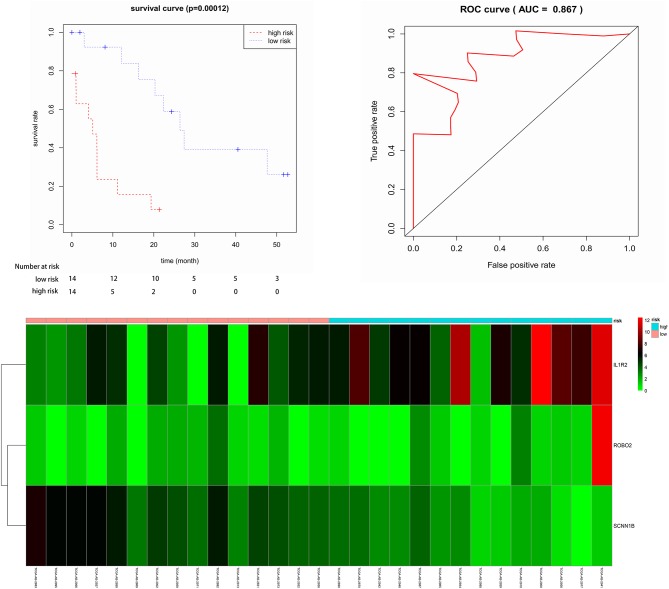
Kaplan-Meier for OS in low-risk and high-risk groups. AUC curve for the risk score, heat map for 6 mRNAs expression level and survival status in all 29 patients.

Twenty-nine elderly CN-AML samples with DNA methylation data and RNA sequencing from TCGA database were divided into the hypermethylated and hypomethylated groups using 8.667 as median AQP1 methylated value. We identified 358 DEGs (161 upregulated; 197 downregulated) between the groups. The heat map and volcano plot for DEGs was shown in Figure S4. The up-regulated genes included the following: (1) genes involved in cancer initiation, promotion, migration, and invasion (HMOX1, HOXB5, HOXB6, HOXB7, HOXB8, and HOXB9) (22, 23); (2) genes inducing angiogenesis and immune suppression (HIF3A) (24); and (3) genes correlating with chemotherapy resistance (CD24) (25). The down-regulated genes included the following: (1) an energy metabolism activator—DUSP27 (26); (2) a putative Hepatocarcinogenesis suppressor—CSMD3 (27); and (3) a cell growth signaling pathway suppressor—KIF26A (28). Subsequently, after leaching of DEGs' association with OS via cox's univariate model, we identified 24 negatively related and 25 positively related mRNAs (*p* < 0.05). Finally, 3 genes (ROBO2, IL1R2, and SCNN1B) were identified as prognostic genes by multivariate cox analysis. The risk score was computed via 3 mRNAs' status and their contribution on overall survival denoted through the coefficient of β in cox's multivariate analysis. The risk score equaled to (0.2016 ^*^ ROBO2's status) + (0.1274 ^*^ IL1R2's status) – (0.5365 ^*^ SCNN1B's status). Next, patients were divided into low- and high-risk groups according to the median predictor score. Low-risk patients had significantly improved overall survival (OS) compared with those in the high-risk group (*p* < 0.05, Figure 3). The 3-year-AUC of receiver operating characteristic curve (ROC) of this signature was 0.867 (Figure 3). In addition, a heat map was constructed to assess the signature, showing that the majority of deaths was in high risk group and demonstrated worse overall survival (Figure 3). These results suggested that the 3-mRNA signature may reliably predict the prognosis in elderly CN-AML patients.

**Figure 4:**
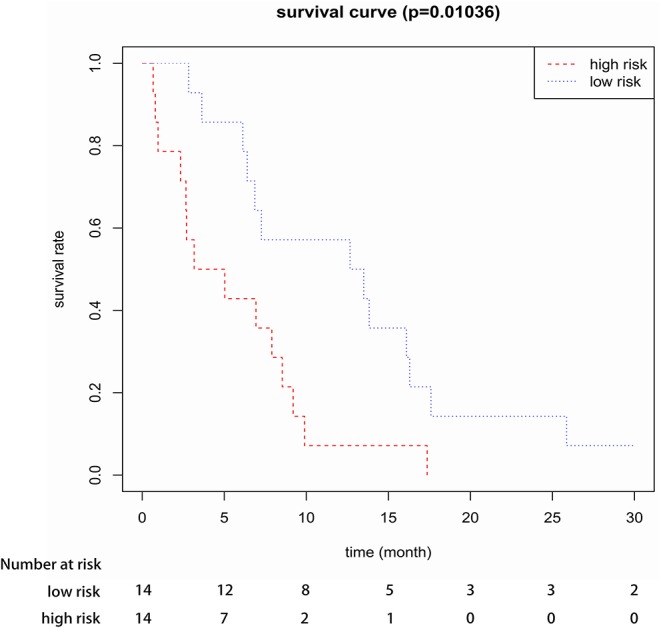
Kaplan-Meier survival analysis of the three-gene prognostic signature in validation data sets (GSE23312).

To confirm the robustness of the 3-gene prognostic signature, we validated our model in another independent dataset of 28 elderly CN-AML patients (age > 60) from GEO database (GSE23312) using the Kaplan-Meier and Cox analyses. The results again showed that the patients in the high-risk group presented a significant shorter survival compared to the low-risk group (Figure 4).

We further analyzed prognostic significance of the model in different European Leukemia Net (ELN) risk subgroups to validate the prognostic value of this three-gene prognostic signature. In the ELN Intermediate-I group, low-risk patients had significantly improved overall survival (OS) compared with those in the high-risk group (Figure S5). However, the model was not tested within the ELN favorable or poor group due to limited patient number with methylation data of the groups (*n* = 2 and *n* = 7, respectively).

### Comparison Between 3-mRNA Prognostic Signature and Other Clinical Prognostic Parameters, Permutation, and Leave-One-Out Cross Validation

We correlated some clinical features with the risk score of the 3-mRNA signature. We found that the risk score was linked to WBC at diagnosis, while it was independent of gender, age, peripheral blasts, bone marrow blasts, platelets count, FLT3 mutation, IDH1 mutation, and NPMc mutation (Figure S6). Cox's univariate model was carried out to explore the relationship between clinical parameters and prognosis. In our study, gender, age at diagnosis, peripheral blasts, bone marrow blasts, platelets count, FAB classifications and IDH1 Mutation could not predict prognosis, while FLT3 mutation was significantly related to survival (Table S1). After adjusting for FLT3 mutation in multivariate analysis, the effect of the prognostic signature kept independent (*p* = 0.003). Next, since the expression of various genes differs with age, we further assessed the expression level of each one of the three AQP1 DNA methylation-associated genes in 3 different >60 age subgroups, including 60–65, 66–70, and 71–75 years old (Figure S7, GEO No: GSE22778). The results shown that ROBO2 expression tends to increase with age. But there was no significant difference in the expression level of IL1R2 and SCNN1B in different age subgroups. In addition, we further analyzed prognostic significance of 3-mRNA prognostic signature in different >60 age subgroups (Figure S8). The results shown that the three-gene prognostic signature based on three AQP1 DNA methylation-associated genes could realize a robust and specific risk stratification for elderly CN-AML patients in different age subgroups. A limitation that should be noticed is the sample size of patients is small when patients are divided into different age subgroups.

Leave-one-out cross validation test (LOO-CV) and permutation test are of great power in appraising the performance of a model, and they are applied to check whether the 3-mRNA signature was able to forecast prognosis of elderly CN-AML patients. Permutation test demonstrated that the AUC of stochastic systems was significant in the group we studied (*P* = 0.0007, Figure S9). LOOCV implied an AUC of 0.834, which proves the 3-gene signature works well in predicting the prognosis of elderly patients with CN-AML.

### Specificity of the 3-mRNA Prognostic Signature in Elderly CN-AML Patients

**Figure 5:**
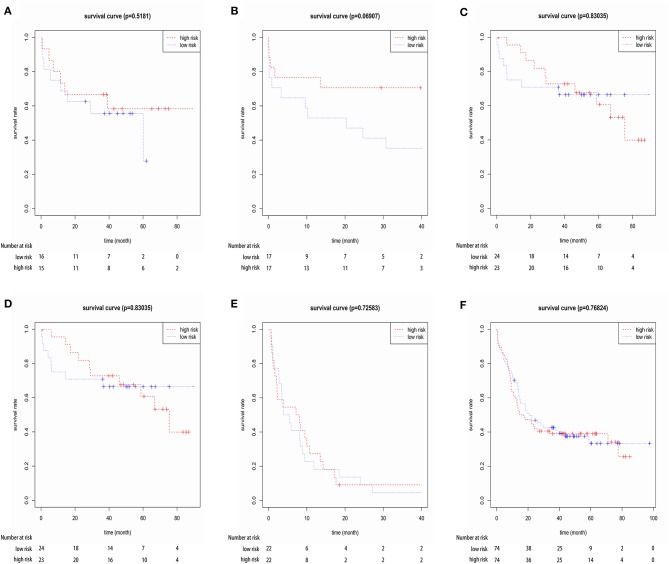
Kaplan-Meier survival analysis of the three-gene prognostic signature in four independent datasets from GEO database (GSE16432, GSE23312, GSE3224, and GSE22778), including 148 samples of younger CN-AML patients (age < 60) and 169 samples of the other 5 common AML subtypes, namely *t*_(8,21)_, *t*_(15,17)_, inv16, +8 and complex karyotype. **(A)** Dataset GSE16432- *t*_(8,21)_, **(B)** dataset GSE23312- *t*_(15,17)_, **(C)** Dataset GSE3224- inv16, **(D)** dataset GSE16432- +8, **(E)** dataset GSE22778- complex karyotype, **(F)** dataset GSE22778- younger CN-AML patients (age < 60).

Furthermore, we investigated the specificity of the prognostic model for elderly CN-AML patients (age > 60). We implemented our model in four independent datasets from GEO database (GSE16432, GSE23312, GSE3224, and GSE22778) and an independent dataset from Target database via Kaplan-Meier and Cox analyses, including 169 samples of 5 other commonly seen AML subtypes, namely *t*_(8,21)_, *t*_(15,17)_, inv16, +8, complex karyotype, as well as younger CN-AML patients (age < 60). The results showed that the prognostic model could not predict the outcome of the other 5 AML subtypes or younger CN-AML patients (Figure 5 and Figure S10), suggesting that it may be used as predictive classifiers with high efficiency for elderly CN-AML patients.

### Functional Enrichment Analysis and PPI Network of DEGs

To evaluate the functional significance of the DEGs associated with AQP1 methylation in elderly CN-AML patients (age > 60), the 358 DEGs were further analyzed by Gene Ontology (GO) enrichment analysis and KEGG pathway analysis, where upregulated and downregulated genes were analyzed, respectively. The most enriched GO terms by upregulated transcripts included “plasma membrane,” “integral component of membrane,” “extracellular region,” “cell adhesion,” and those by downregulated transcripts included “integral component of membrane,” “plasma membrane,” “calcium ion binding,” and “signal transduction” (Figure S11). KEGG pathway analysis showed 12 pathways associated with upregulated transcripts and the most enriched network was “Neuroactive ligand-receptor interaction.” Pathway analysis also showed 8 pathways related with downregulated transcripts and “Pathways in cancer” was the most enriched network (Figure S11). Of all these pathways, “ECM-receptor interaction” and “Tyrosine metabolism” have been reported to be important causes of tumor metastasis and invasion (29, 30).

In addition, the protein–protein interaction (PPI) network was made up of 99 nodes and 144 edges (Figure S12A). Furthermore, 12 hub genes were recognized in the network when the cut-off criterion was set to be “Degrees≥5” (Figure S12B). Among them, CXCL10 gene has been reported to promote cell growth, metastasis and decrease cell apoptosis in chronic myeloid leukemia (31); The high C5AR1 expression is related to shorter overall survival and increased bone metastasis in lung tumors (32); FPR1 gene plays a key role in the mechanism of cellular drug resistance in acute lymphoblastic leukemia (33); LHCGR gene expression is correlated with adrenocortical tumorigenesis (34). The discovery of these genes may provide us with an opportunity to treat elderly CN-AML patients using gene targeting agents.

### Association Between Genome-Wide lncRNA, microRNA Profiles, and AQP1 Methylation

To further assess the mechanism of AQP1 methylation in elderly CN-AML, we derived AQP1 methylation-associated lncRNA and microRNA expression profiles by microarray analysis. We analyzed 29 elderly CN-AML samples with DNA methylation data and RNA sequencing from TCGA database and identified 41 up-regulated and 33 down-regulated lncRNAs which were significantly related with AQP1 methylation (*P* < 0.05), the heat map and volcano plot for different lncRNAs were shown in Figure S13. The up-regulated lncRNAs included lncRNAs involved in chemotherapy resistance (MIR100HG and MGC32805) (35, 36) and tumorigenesis promoters (AC011632.1 and LINC00355) (37, 38), while down-regulated genes included MAGI2-AS3, an inhibiting factor of breast cancer cell growth via the Fas/FasL signaling pathway (39). Kaplan-Meier method was carried out to explore their association with prognosis. 6 lncRNAs (AC099552.2, GNA14-AS1, KC6, LINC00355, LINC01482, and LINC02139) were found significantly associated with OS of elderly CN-AML patients, and all of them were negatively related to OS (*p* < 0.05, Figure S14).

Furthermore, 20 elderly CN-AML samples with DNA methylation data and miRNA expression from TCGA database were analyzed. Six up-regulated and 2 down-regulated miRNAs which were significantly related with AQP1 methylation (*P* < 0.05) were identified. The heat map and volcano plot for different miRNAs were shown in Figure S15. The down-regulated MiR-577 have been reported previously to have important tumor-suppressive properties. MiR-577 targets tumor-promoting gene WNT2B which mediates Wnt/β-catenin pathway to suppresses cell proliferation and epithelial-mesenchymal transition in non-small cell lung cancer (40). Some of the up-regulated miRNAs serve as potential oncogenes in carcinogenesis (hsa-mir-452) (41), and some promote tumor cell proliferation and inducing immune escape (hsa-mir-224) (42).

### Identification of CpG Sites of AQP1 Associated With AQP1 DNA-Methylation and Prognosis of Elderly CN-AML

**Figure 6:**
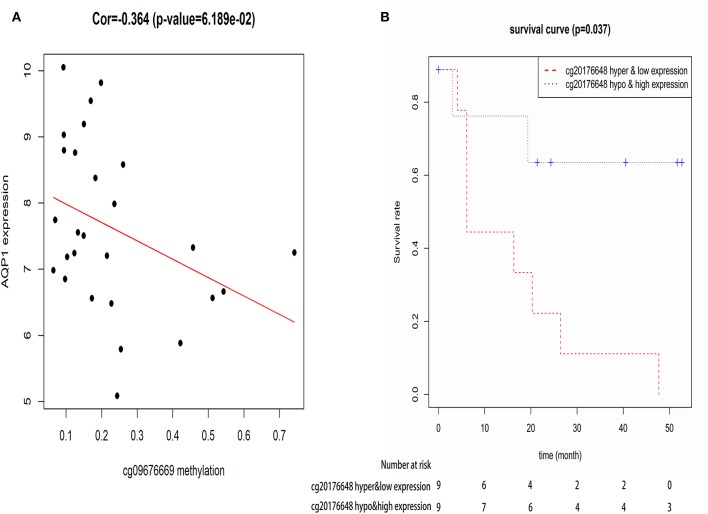
The correlation between CpG site of AQP1-cg09676669 and AQP1 methylation level **(A)**, and the correlation between CpG site of AQP1-cg09676669 and the overall survival of elderly CN-AML patients **(B)**.

Recent advances have facilitated the screening of CpG sites at a genomic level by whole genome screening technologies, giving a more thorough view of the methylation landscape (43). As one gene contains multiple CpGs, Pearson correlation coefficients were calculated to identify actual CpG sites of AQP1 whose expression levels were affected by DNA methylation. The results showed that 8 CpG sites of AQP1 (cg00516678, cg00622010, cg07135629, cg09676669, cg10132917, cg11827925, cg18307978, and cg20176648) were regulated by DNA methylation. Subsequently, these 8 CpG sites of AQP1 were added to cox's univariate model to identify CpG sites of AQP1 related with OS. We found that cg09676669 related with OS and DNA methylation, thus acting as a potential diagnosis and prognosis biomarker (Figure 6).

## Discussion

Elderly patients with CN-AML make up the largest group of all primary AML. However, other than clinical trials, therapy options provided for elderly AML especially elderly CN-AML patients still remain limited (44, 45). On the basis of patient-specific (46) and AML-related prognosis factors (47), AML patients are divided to take intensive chemotherapy (IC), low-intensity therapy (LIT), and best supportive care (BSC) (48). Although scoring systems have been proposed to rationalize medical decisions, there are still large variations in clinical practice (48) and high heterogeneity in patients' prognosis, which underlines paucity of evidence supporting medical decisions. It is urgent to identify molecular mechanisms associated with progression and prognosis of the disease to help us better understand the pathogenesis of elderly CN-AML and improve patients' outcome. The previous study has shown that ITPR2 (49) and ATP1B1 (50), which are responsible for Ca2+, K+ and Na+ transport, are predictive of poor outcomes in CN-AML. AQP1, a transmembrane water channel protein, has shown promising and independent prognostic values in solid tumor (51), while its effect on the clinical outcomes in elderly CN-AML was not clear.

We found that AQP1 expression was down-regulated in the bone marrow of CN-AML compared with normal BM. Furthermore, the high expression and hypomethylation of AQP1 was associated with longer OS in elderly CN-AML. To further understand the function of AQP1 methylation in the pathogenesis and prognosis of elderly CN-AML, we conducted a multi-omics analysis exploring AQP1 DNA methylation-associated mRNAs, miRNAs, lncRNAs, methylation loci, and cell signaling pathways. These alterations of transcriptomes may have mediated the mechanisms underlying the correlation between AQP1 methylation and the prognosis of elderly CN-AML. While there are no novel drugs targeting AQP1 DNA methylation-related genes, there are demethylation drugs such as DNA methyltransferase inhibitors [5-azacytidine (52) and 5-aza-2′-deoxycytidine (53)], second-generation DNMT inhibitors [Zebularine (54) and Guadecitabine (55)], and lysine demethylase inhibitors (56), and drugs that affect the expression level of AQP1 like Ziziphora clinopodioides which upregulates aquaporin 1 (57). It is worth noting that drugs affecting AQP1 expression are not currently used for cancer treatment.

It's worth noting that most studies show that AQP1 gene acts as an oncogene in various solid cancers to promote cancer development (12–14), whereas only a few studies report AQP1 as a tumor suppressor that inhibits tumor growth (21, 58). Recently, Marietta Tan et al. also showed that AQP1 was epigenetically downregulated by promoter methylation and was associated with improved prognosis in salivary gland adenoid cystic carcinoma (20). While we show that AQP1 gene acts as a tumor suppressor gene whose high expression and hypomethylation were associated with good prognosis in elderly CN-AML patients (age > 60 years). These data indicate that AQP1 may perform different functions in tumorigenesis. Therefore, more studies are needed to explore the specific roles and mechanisms of AQP1 in different types of cancer.

The most important feature of this study was the establishment of a three-gene prognostic signature based on three AQP1 DNA methylation-associated genes, which may realize a robust and specific risk stratification for elderly CN-AML patients. This prognostic model had 3 features. Firstly, this model had high sensitivity, and could accurately predict the prognosis of patients. The prognostic value of the 3-mRNA signature was validated by Permutation test and LOO-CV which are of great power to assess the performance of a model. The highest AUC value (0.867) of 3-year ROC of this model also indicated that it provided the best prognostic function. Secondly, this model is suitable for different patient groups. The universality of this model's prognostic value was supported by an independent cohort (GSE23312). Thirdly, this model is highly practical in that with three genes in the signature, their expression levels could be measured by relatively inexpensive PCR-based technology to achieve prospective risk stratification of individual patients. Moreover, the specificity of the three-gene prognostic signature to elderly CN-AML suggests that the underlying molecular mechanisms and pathogenesis may differ between young and elderly AML and different leukemia subtypes. More research is needed to explore the underlying mechanisms.

Importantly, we identified an AQP1-specific methylated site cg09676669 as a potential diagnostic and prognosis biomarker for elderly CN-AML patients. Therefore, by examining the methylation level of the site cg09676669 in the peripheral blood or bone marrow through Bisulfite sequencing PCR (BSP), we can preliminarily predict the prognosis of elderly CN-AML patients.

It should be noticed that NPM1 mutation was reported to be associated with better prognosis, especially in normal karyotype AML (59). However, the impact of NPM1 mutations on overall survival in *de novo* AML is controversial. A recent study showed that NPM1 mutations may promote the expression of HOXA5, HOXB5, HOXA10, PBX3, and MEIS1 in AML cells, which was correlated with a worse prognosis in AML (60). These results were consistent with previous findings showing that the gene expression profile of NPM1c mutated AML cells is characterized by upregulated genes involved in stem cell maintenance (61). In our study, NPM1 mutation was associated with AQP1 hypermethylation, which predicts worse outcome. It is worth noticing that the many patients with missing or unknown mutation information in the TCGA database we used may have complicated our analysis of the effects of NPM1 mutation.

## Conclusion

Taken together, we demonstrate that AQP1 hypomethylation and high expression have prognostic significance for elderly CN-AML patients (age > 60). Moreover, our genome-wide analysis of abnormal gene, lncRNA, signaling and miRNA expression associated with AQP1 methylation may help understand the role of AQP1 in elderly CN-AML and develop new therapeutic strategies. Importantly, we developed a three-gene panel based on genes associated with AQP1 DNA methylation to predict cancer risk and the prognosis of elderly CN-AML patients.

## Data Availability Statement

Publicly available datasets were analyzed in this study. This data can be found here: The Cancer Genome Atlas (TCGA) and GEO.

## Ethics Statement

This study was conducted in full compliance with the publication guidelines provided by TCGA and GEO. The data were obtained from TCGA and GEO, so the approval of an ethics committee was not needed.

## Author Contributions

XY collected and analyzed the data and wrote the manuscript. HH, SH, AX, FF, SL, HY, and LC researched literature, edited the paper, and revised the manuscript. CS and YH conceived and designed this study, analyzed the data, and wrote the manuscript. All authors have read and approved the manuscript.

## Conflict of Interest

The authors declare that the research was conducted in the absence of any commercial or financial relationships that could be construed as a potential conflict of interest.

## Supplementary Material

Figure S1 Differential expression of AQP1 between younger and elderly CN-AML patients from GSE22778.

Figure S2 The correlation between AQP1 methylation and expression in elderly CN-AML patients from TCGA.

Figure S3 The correlation between AQP1 methylation and clinical features in 39 elderly CN-AML patients from TCGA.

Figure S4 Heatmap **(A)** and volcano plot **(B)** of the differentially expressed genes (DEGs) between AQP1 hypermethylated and hypomethylated groups.

Figure S5 Kaplan-Meier survival analysis of the three-gene prognostic signature in the ELN Intermediate-I group.

Figure S6 The correlation between the risk score of the 3 mRNA signatures and clinical features in 29 elderly CN-AML patients from TCGA.

Figure S7 The differential expression levels of AQP1 DNA methylation-associated genes ROBO2 **(A)**, IL1R2 **(B)** and SCNN1B **(C)** in different age subgroups from GSE22778.

Figure S8 Kaplan-Meier survival analysis of the three-gene prognostic signature in different age subgroups from TCGA. **(A)** Patients aged 60–65, **(B)** patients aged 66–70, and **(C)** patients aged 71–75.

Figure S9 Permutation test for three-gene prognostic signature.

Figure S10 Kaplan-Meier survival analysis of the three-gene prognostic signature in Target database including 149 samples of younger AML patients (age < 30).

Figure S11 Top 20 enrichment of GO terms and pathways for differentially expressed intersection mRNAs associated with AQP1 methylation in elderly CN-AML patients (age > 60) (the bar plot shows the enrichment scores of the significant enrichment GO terms and pathways).

Figure S12 Protein–protein interaction network of differentially expressed intersection mRNAs associated with AQP1 methylation in elderly CN-AML patients (age > 60) **(A)** and 12 hub genes selected from protein–protein interaction network **(B)**. The bar plot shows the enrichment scores of the interactions between the nodes.

Figure S13 Heat map **(A)** and volcano plot **(B)** of the differentially expressed lncRNA between AQP1 hypermethylated and hypomethylated group.

Figure S14 Kaplan-Meier survival curves for 6 lncRNAs associated with overall survival from the differentially expressed lncRNA between AQP1 hypermethylated and hypomethylated group.

Figure S15 Heat map **(A)** and volcano plot **(B)** of the differentially expressed miRNA between AQP1 hypermethylated and hypomethylated group.

Table S1 Univariate Cox analysis of clinical parameters with the prognosis in elderly CN-AML patients.

## Abbreviations

AML: acute myeloid leukemia
lncRNA: long non-coding RNA; The Cancer Genome Atlas
OS: overall survival
miRNA: microRNA
AUC: the area under Receiver Operating Characteristic curve
ROC: receiver operating characteristic curve
LOO-CV: leave-one-out cross validation
DAVID: the database for annotation, visualization and integrated discovery
GO: gene ontology
PPI: the protein–protein interactions
KEGG: Kyoto Encyclopedia of Genes and Genomes
mRNA: messenger RNA.
