# Supplementary figures and images for: A Novel Scoring System for Risk Assessment of Elderly Patients With Cytogenetically Normal Acute Myeloid Leukemia Based on Expression of Three AQP1 DNA Methylation-Associated Genes
Source: Front Oncol. 2020 Apr 21;10:566. doi: 10.3389/fonc.2020.00566 (PMC7186486; doi:10.3389/fonc.2020.00566)

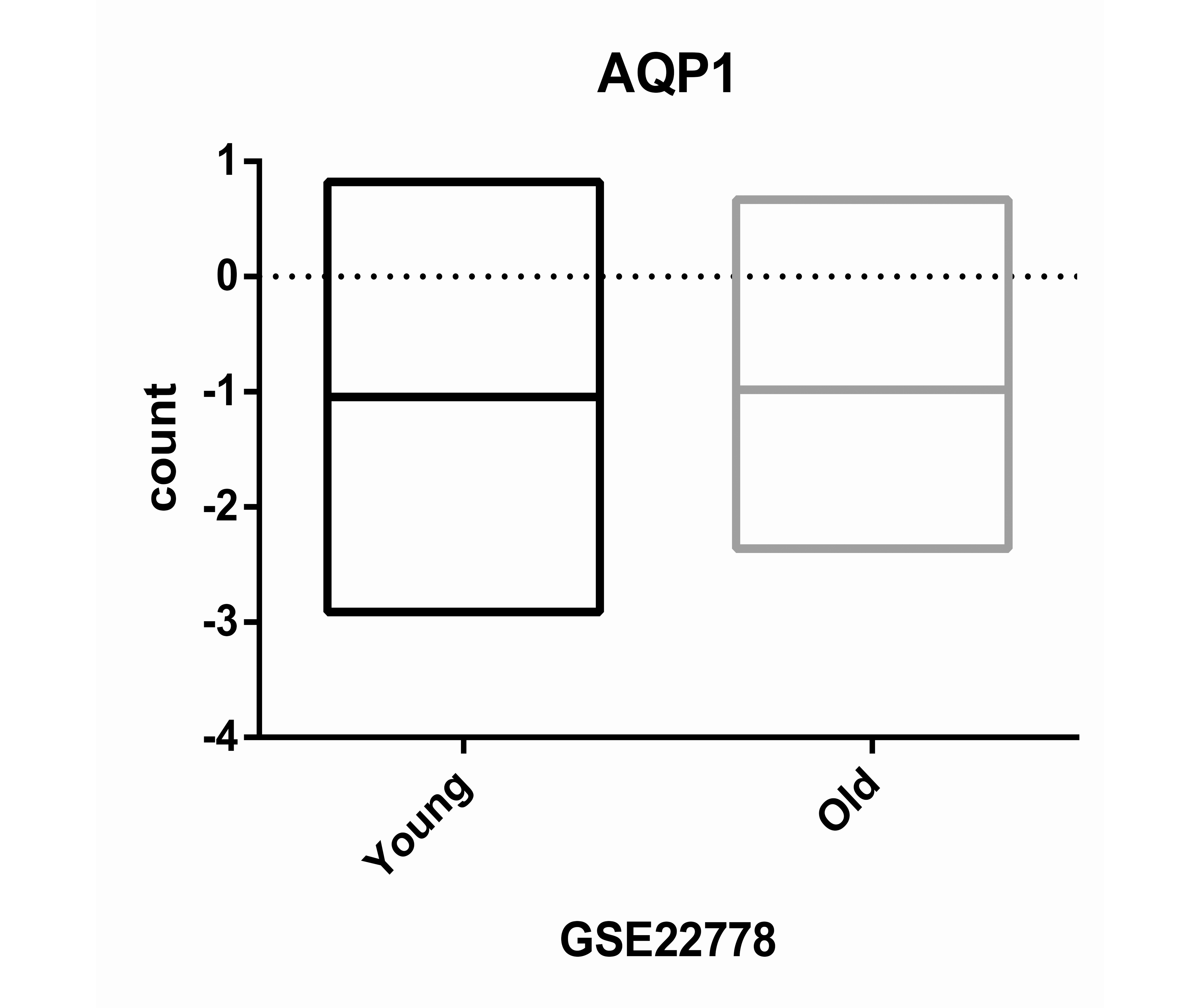

Supplement: Figure S1 — Differential expression of AQP1 between younger and elderly CN-AML patients from GSE22778. [file Image_1.TIF]

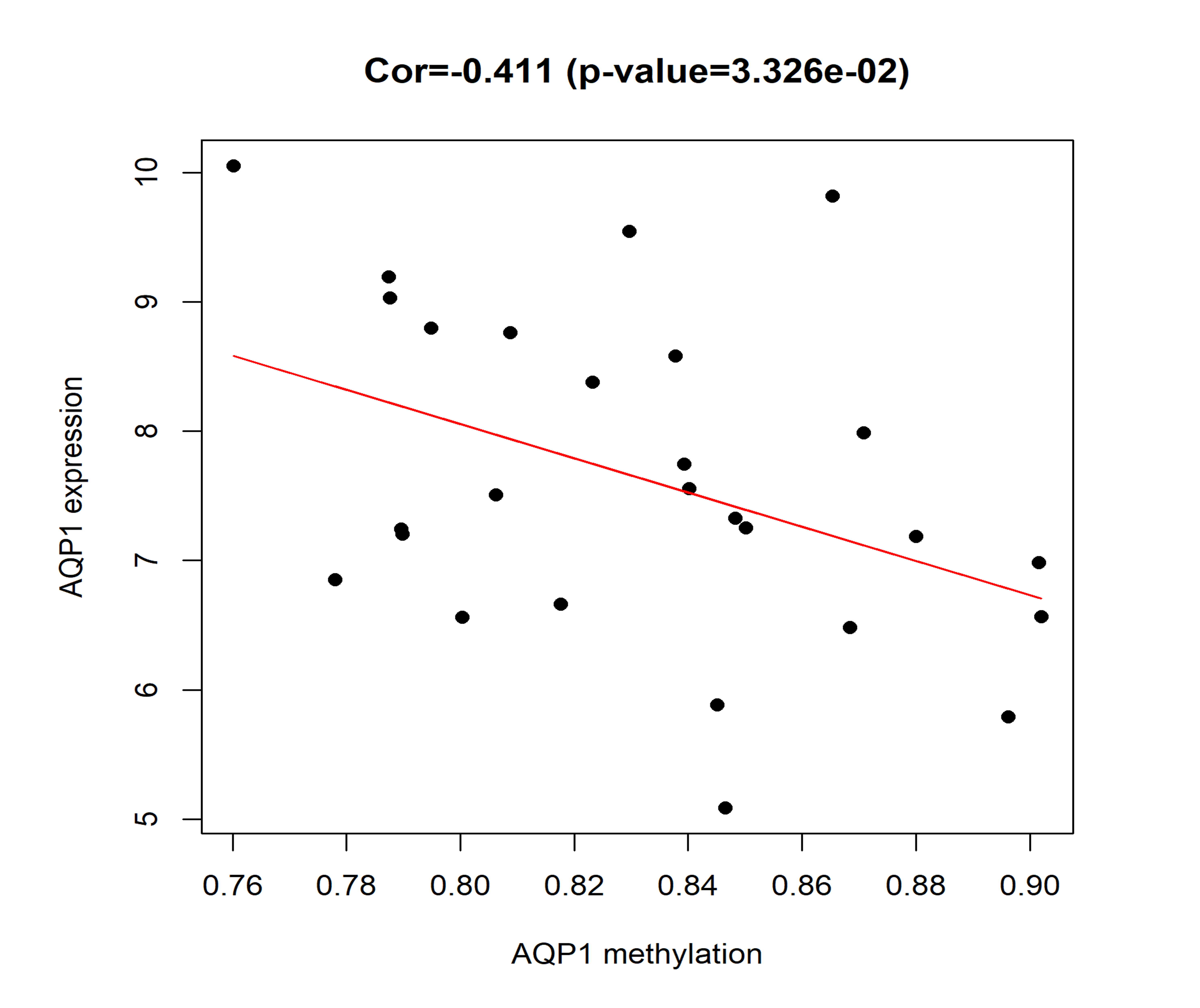

Supplement: Figure S2 — The correlation between AQP1 methylation and expression in elderly CN-AML patients from TCGA. [file Image_2.TIFF]

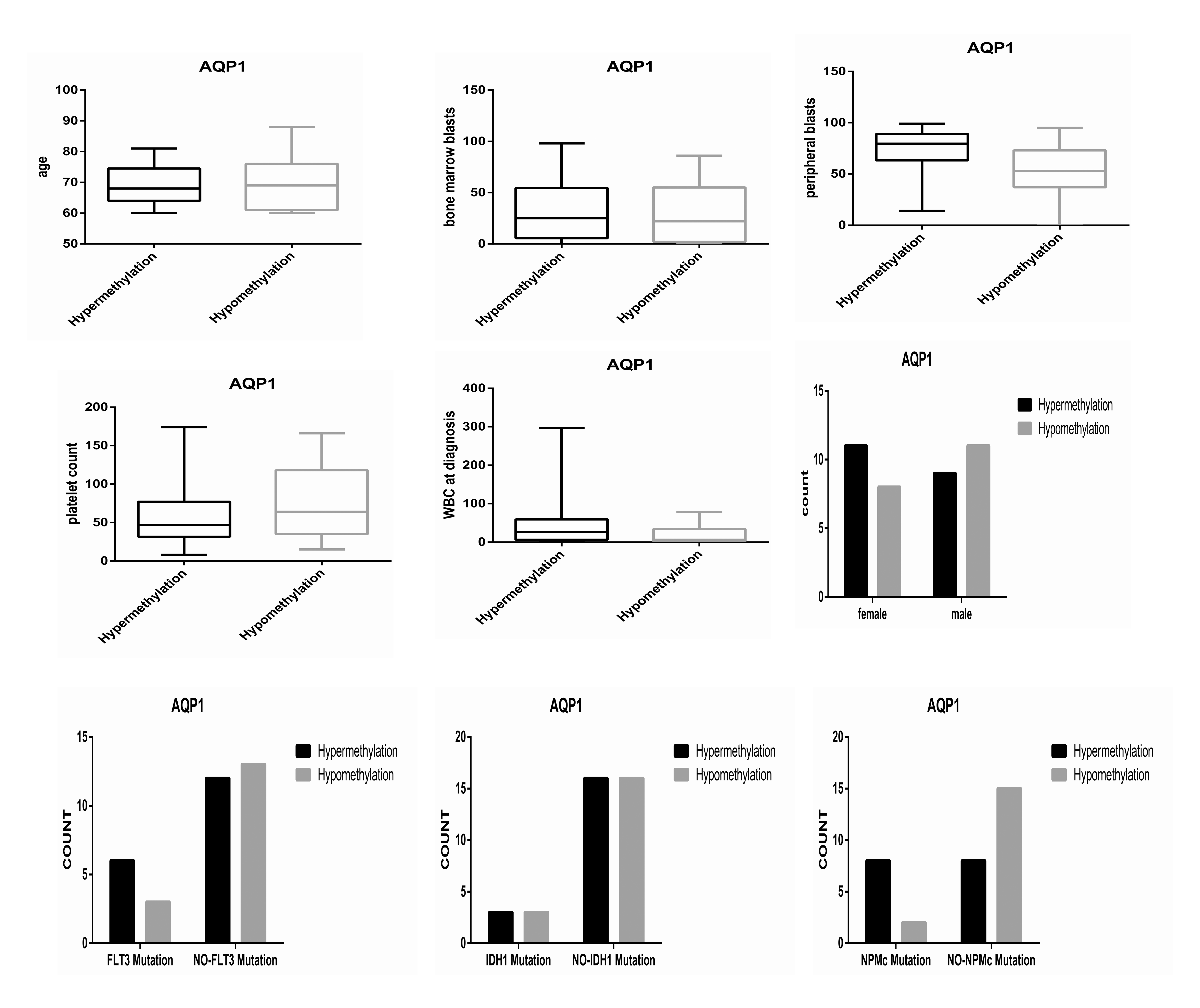

Supplement: Figure S3 — The correlation between AQP1 methylation and clinical features in 39 elderly CN-AML patients from TCGA. [file Image_3.TIF]

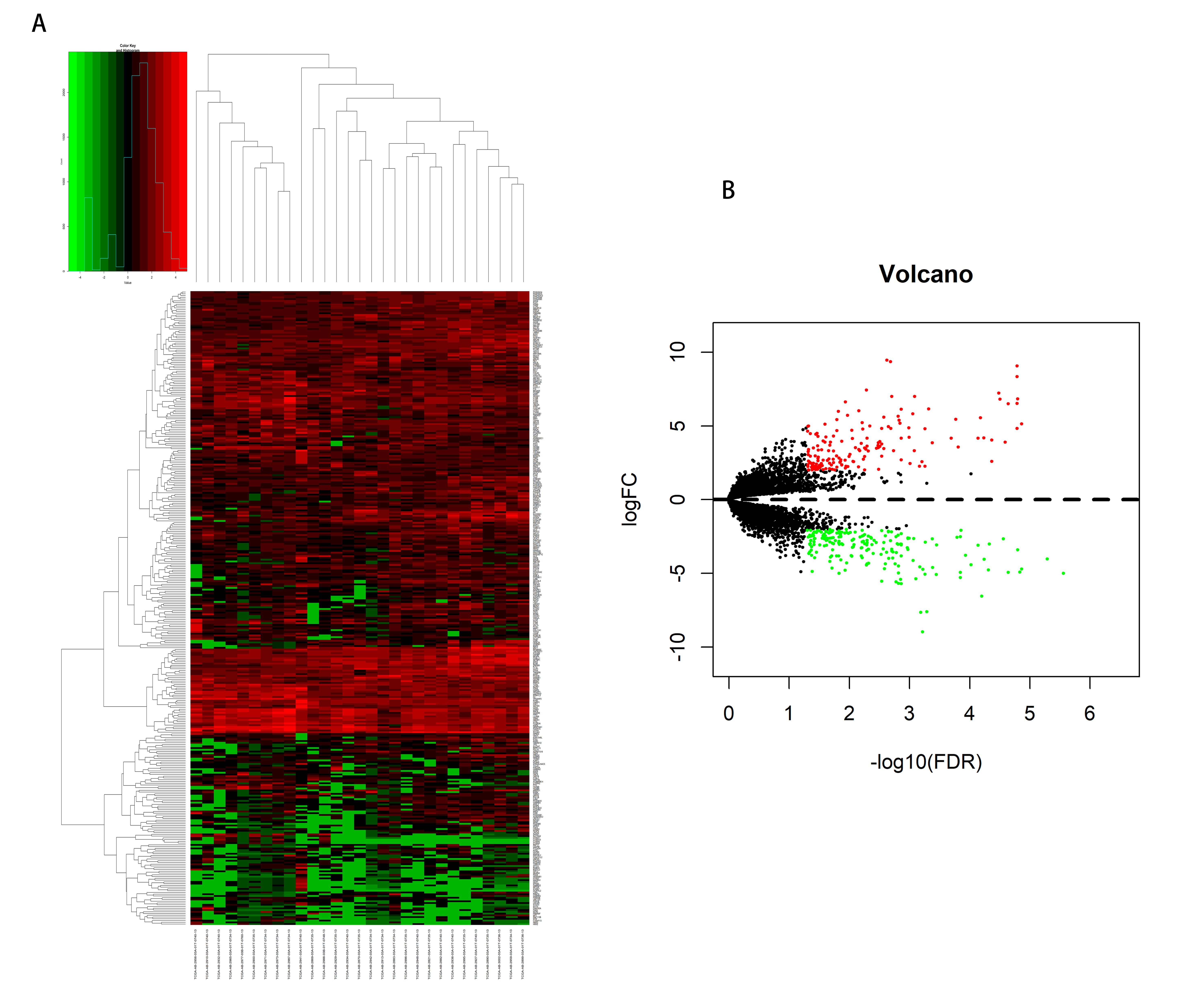

Supplement: Figure S4 — Heatmap (A) and volcano plot (B) of the differentially expressed genes (DEGs) between AQP1 hypermethylated and hypomethylated groups. [file Image_4.TIF]

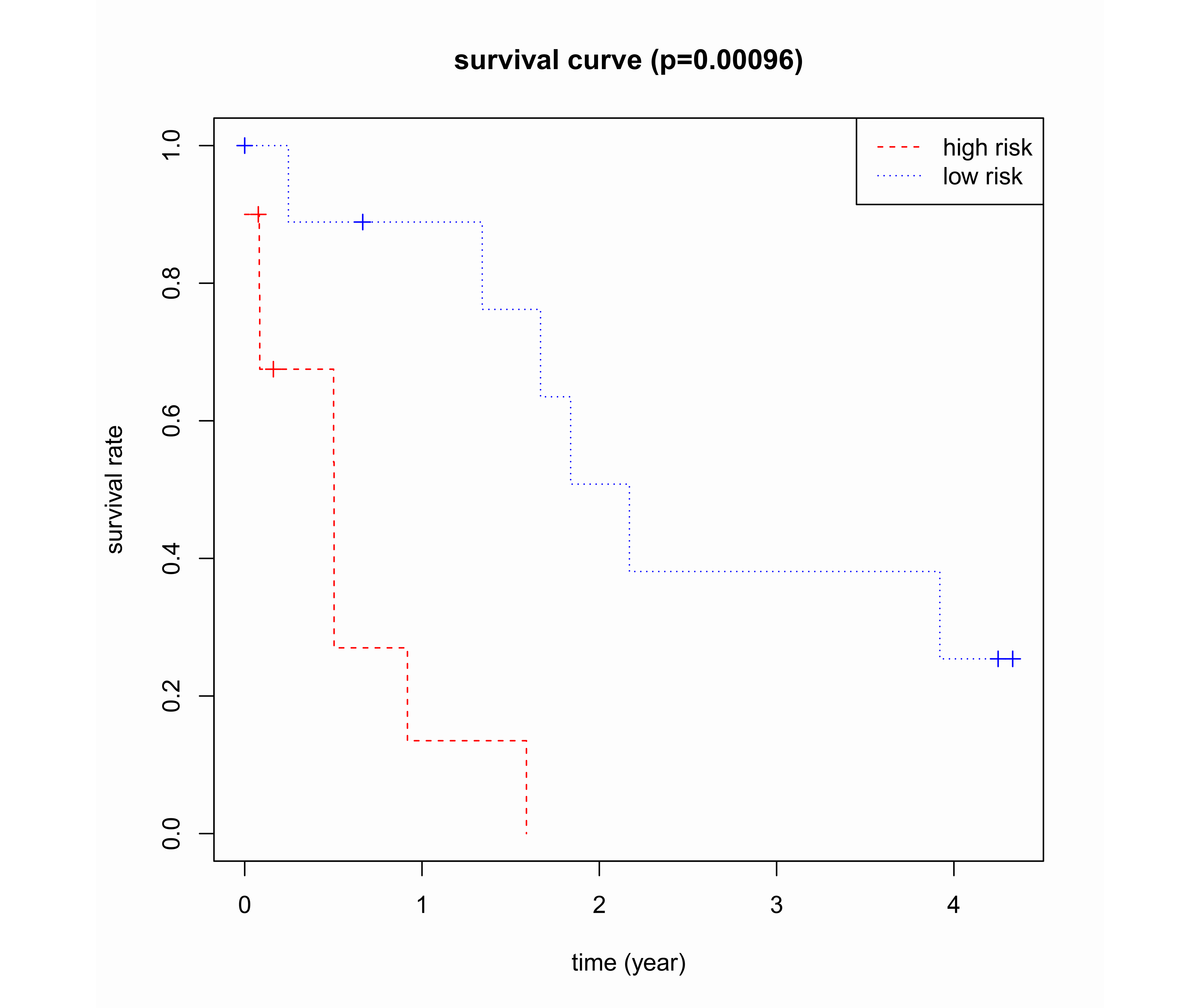

Supplement: Figure S5 — Kaplan-Meier survival analysis of the three-gene prognostic signature in the ELN Intermediate-I group. [file Image_5.TIF]

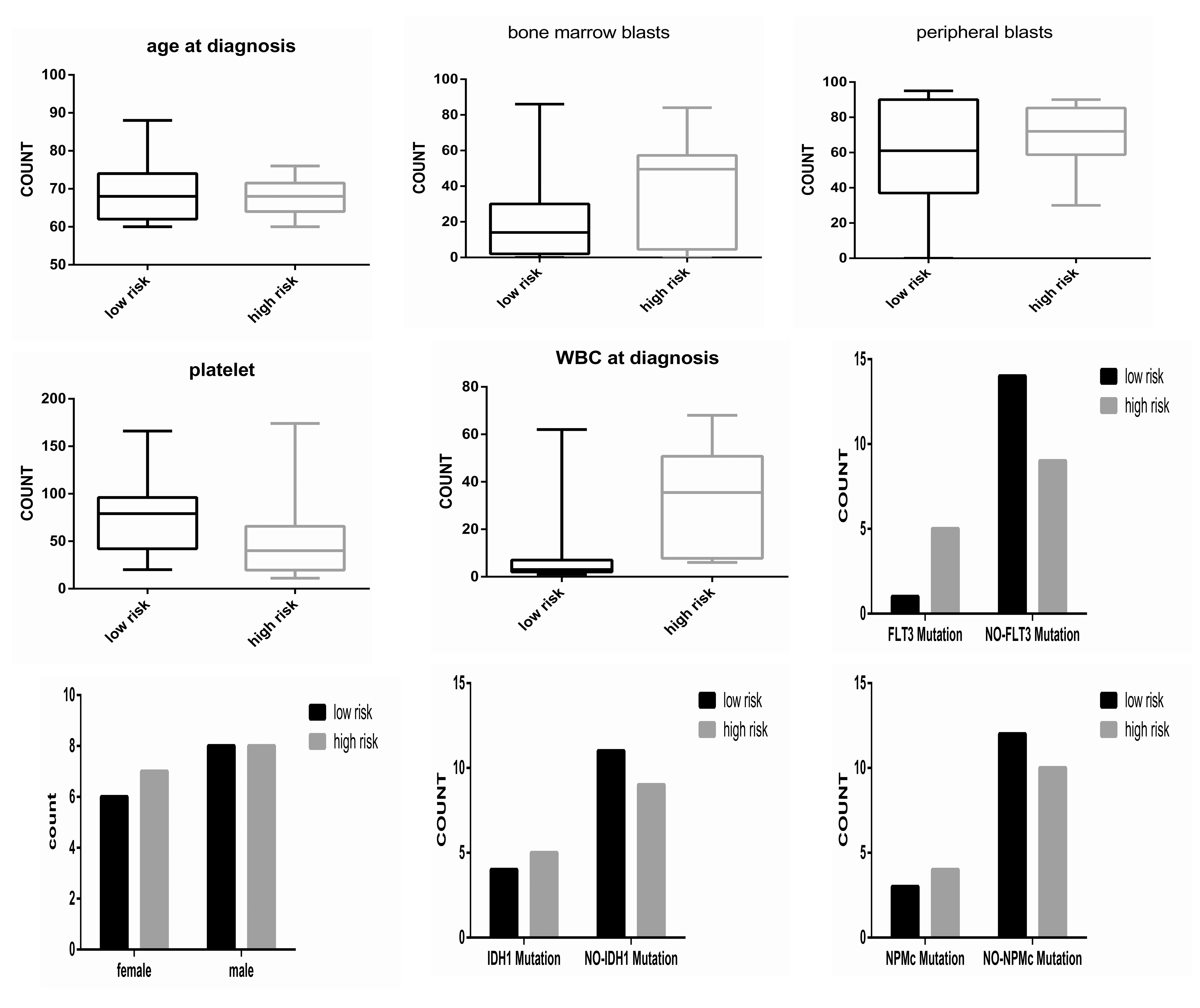

Supplement: Figure S6 — The correlation between the risk score of the 3 mRNA signatures and clinical features in 29 elderly CN-AML patients from TCGA. [file Image_6.TIF]

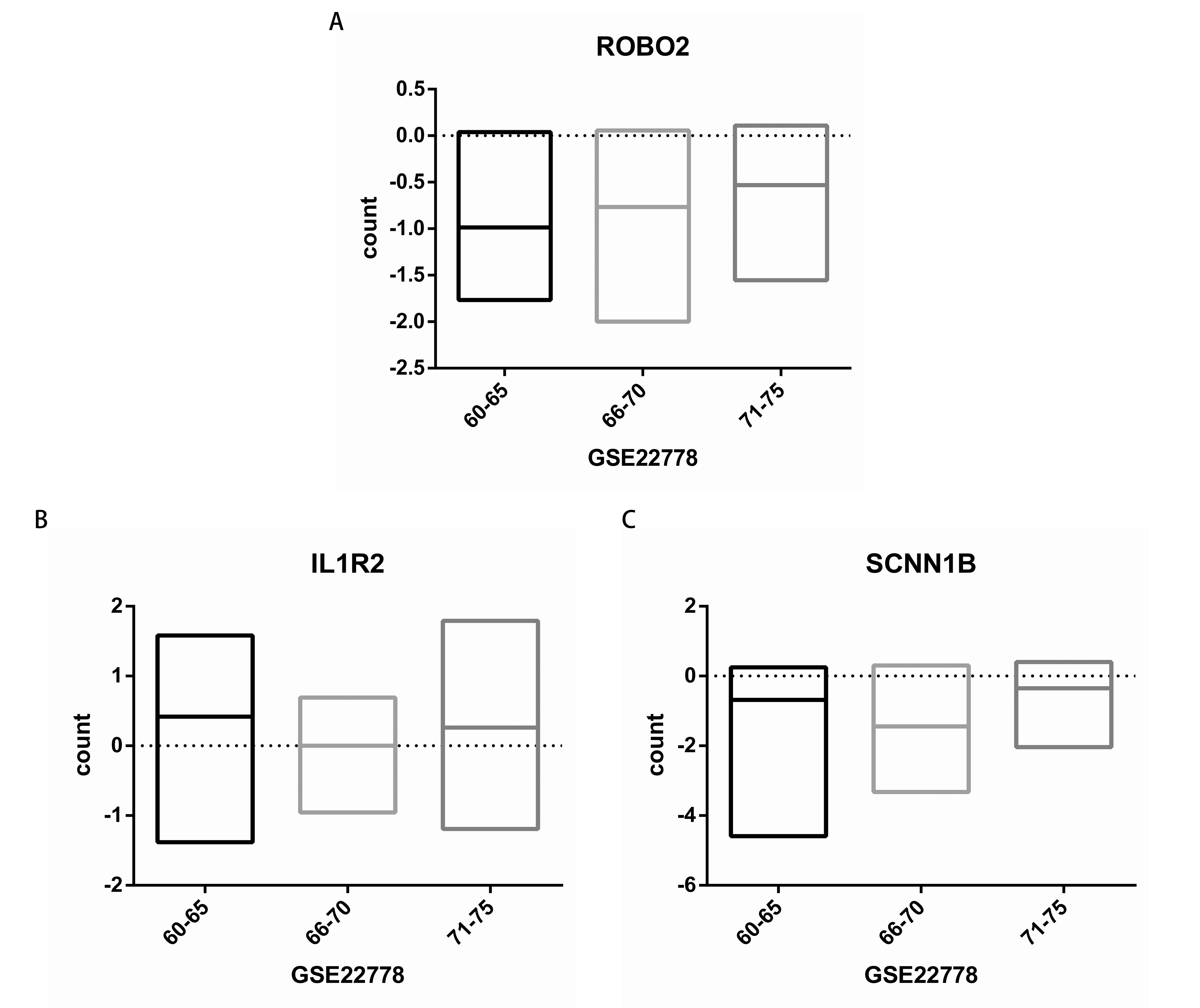

Supplement: Figure S7 — The differential expression levels of AQP1 DNA methylation-associated genes ROBO2 (A), IL1R2 (B) and SCNN1B (C) in different age subgroups from GSE22778. [file Image_7.TIF]

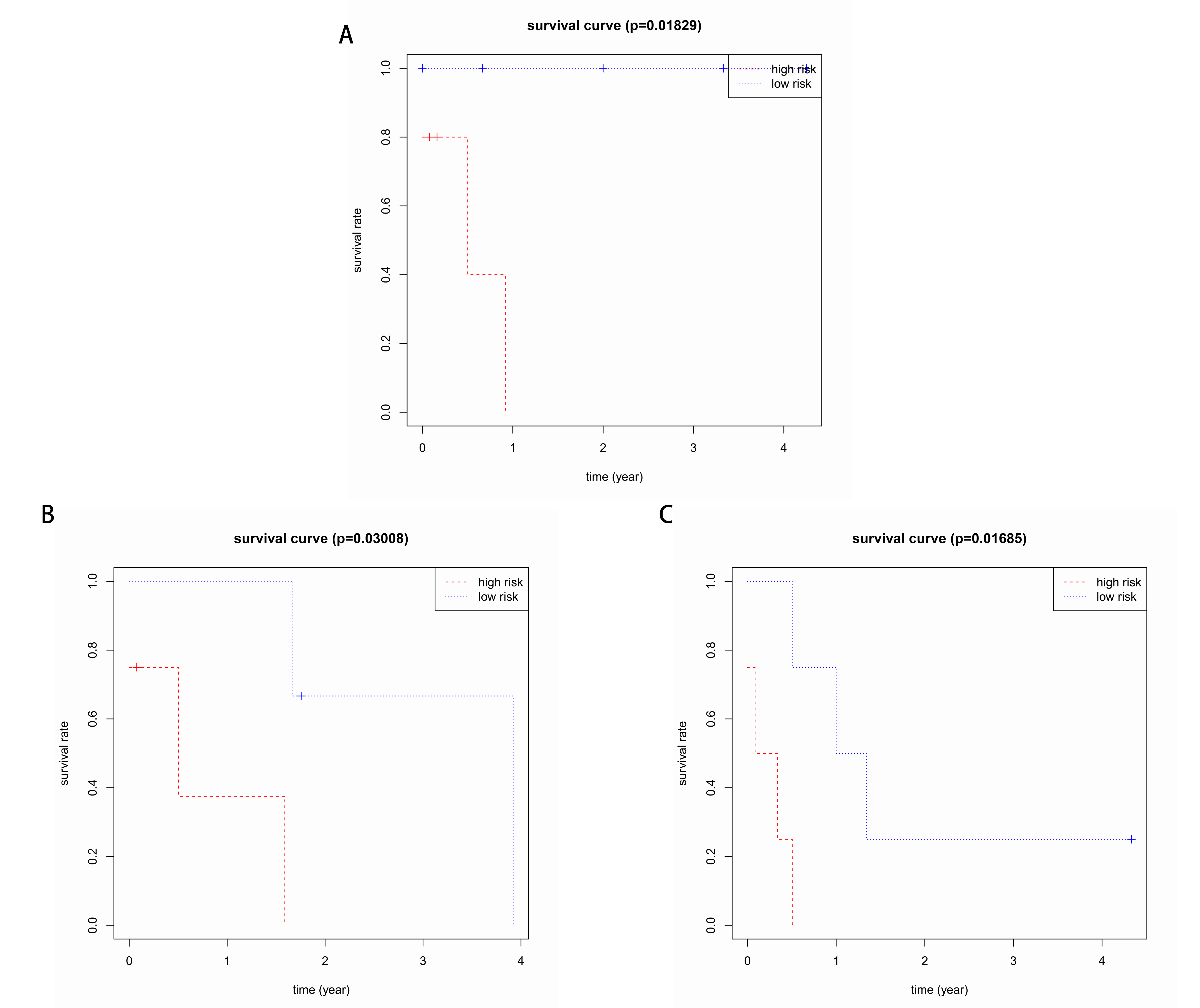

Supplement: Figure S8 — Kaplan-Meier survival analysis of the three-gene prognostic signature in different age subgroups from TCGA. (A) Patients aged 60–65, (B) patients aged 66–70, and (C) patients aged 71–75. [file Image_8.TIF]

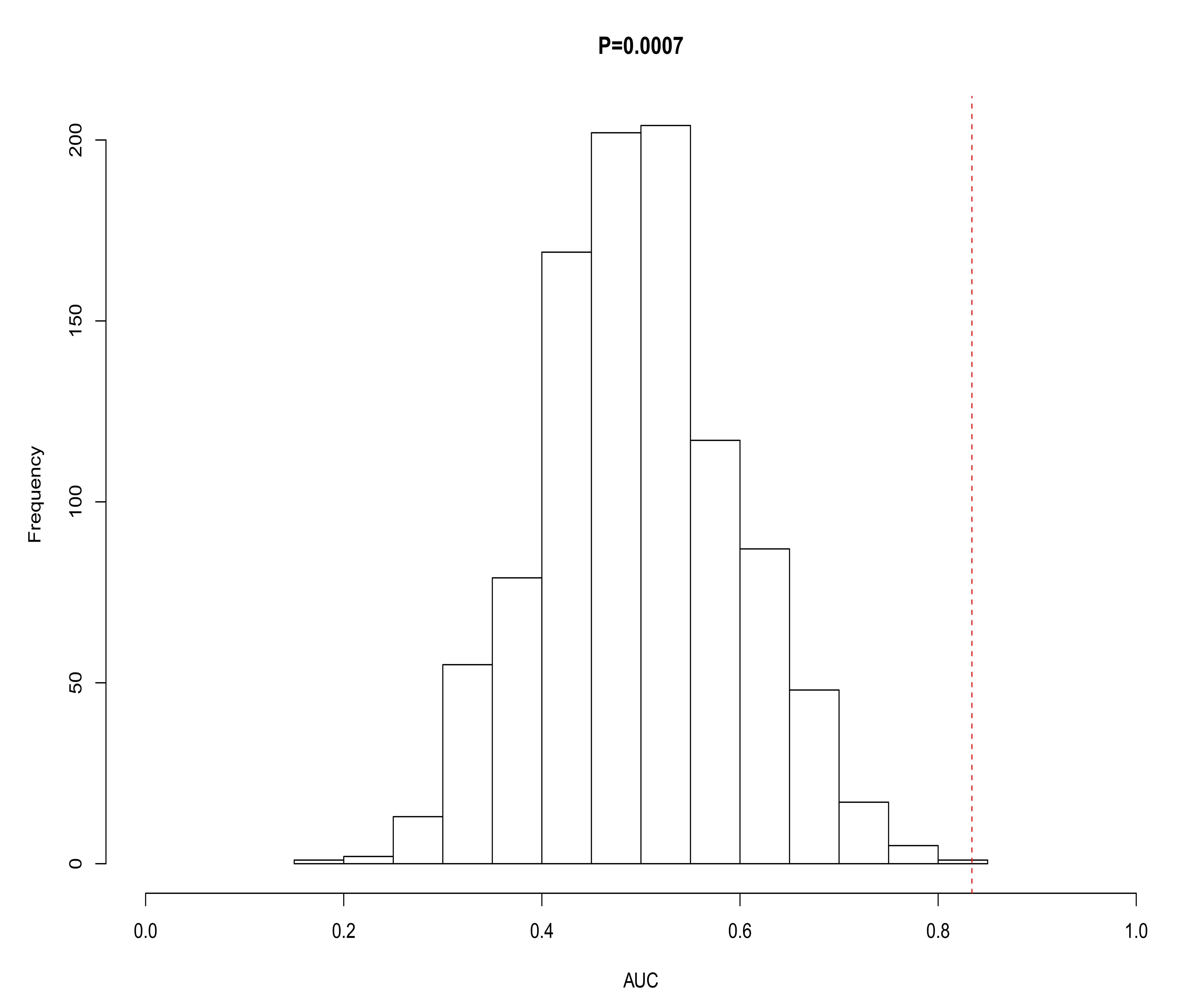

Supplement: Figure S9 — Permutation test for three-gene prognostic signature. [file Image_9.TIF]

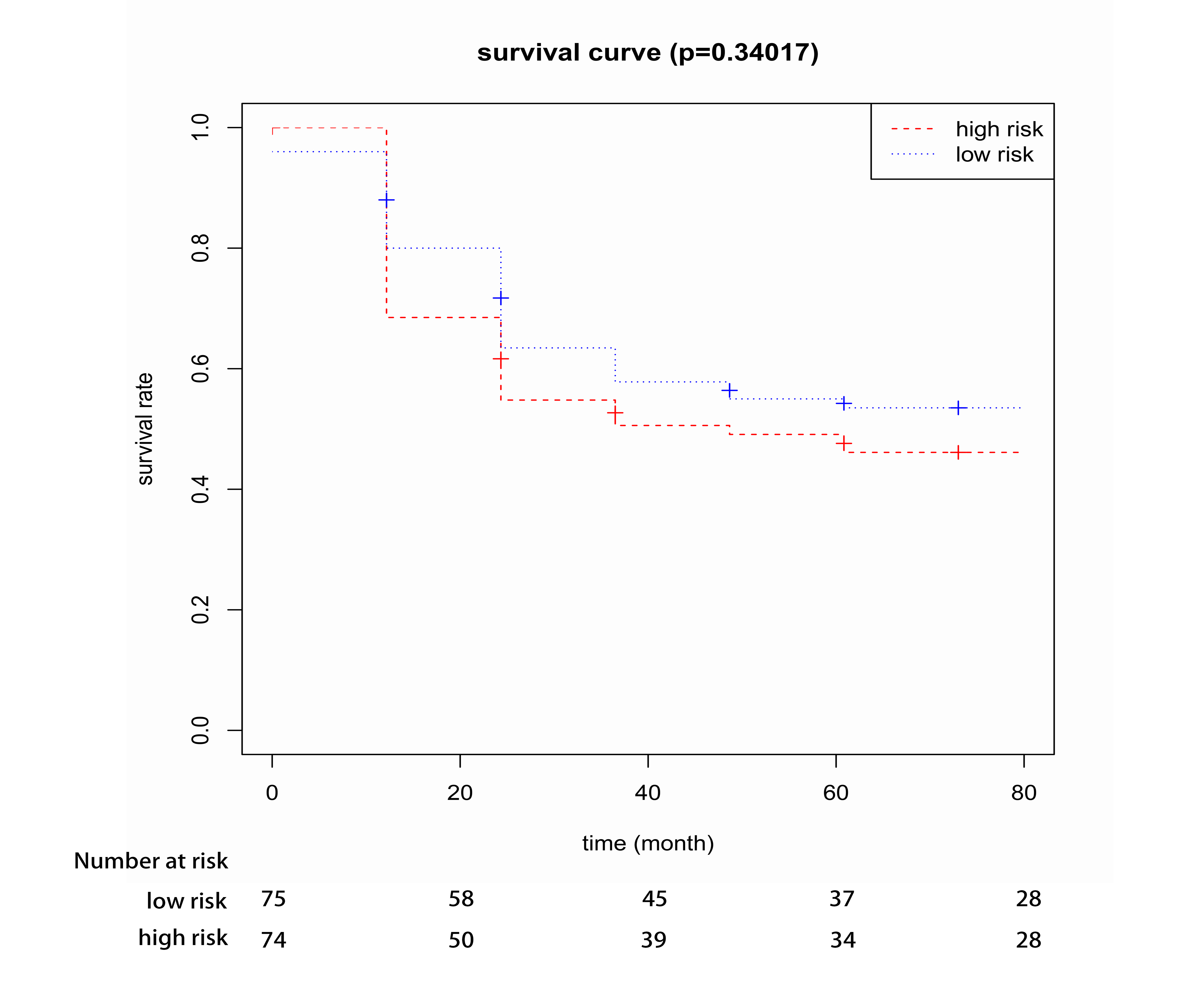

Supplement: Figure S10 — Kaplan-Meier survival analysis of the three-gene prognostic signature in Target database including 149 samples of younger AML patients (age < 30). [file Image_10.TIF]

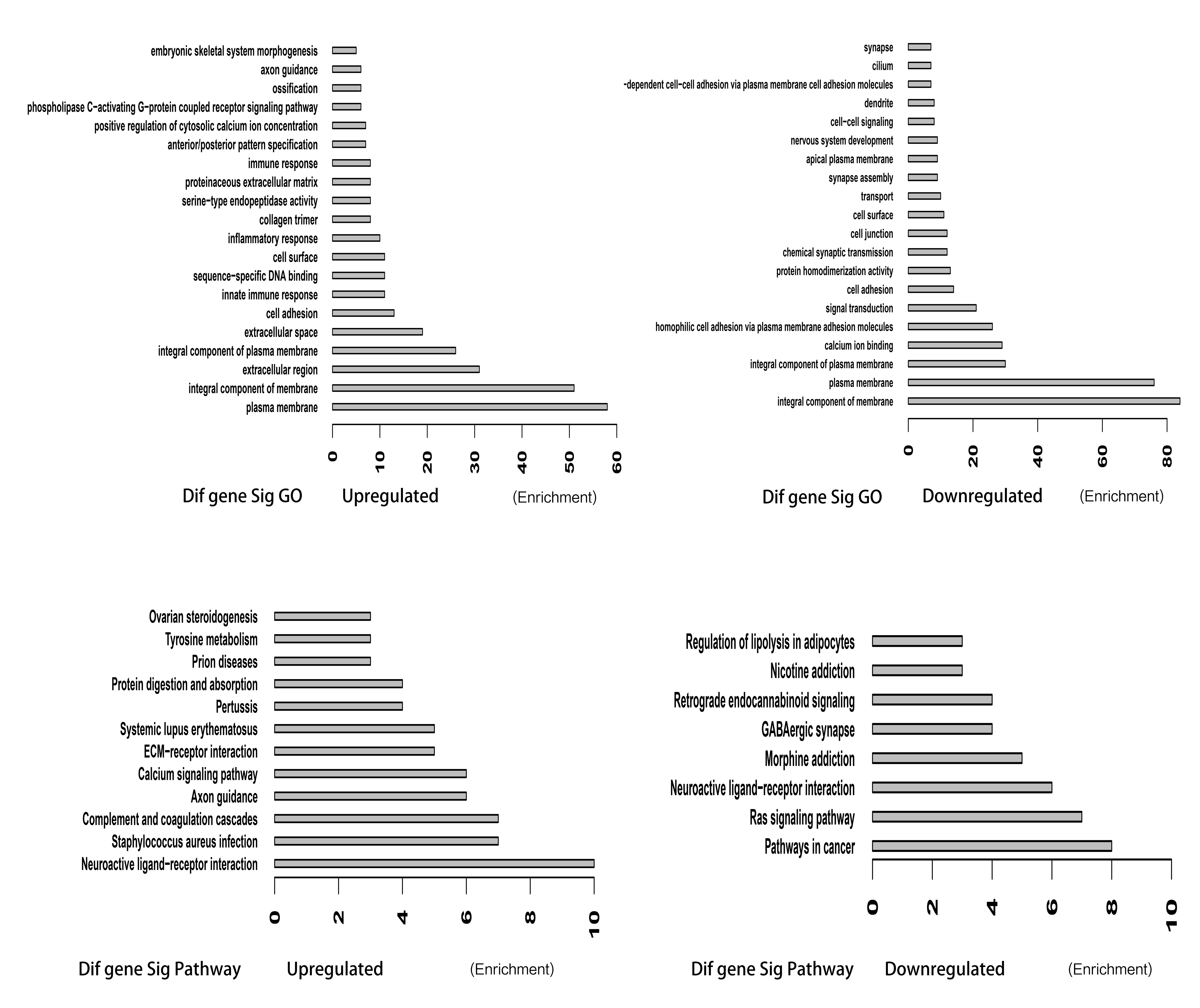

Supplement: Figure S11 — Top 20 enrichment of GO terms and pathways for differentially expressed intersection mRNAs associated with AQP1 methylation in elderly CN-AML patients (age > 60) (the bar plot shows the enrichment scores of the significant enrichment GO terms and pathways). [file Image_11.TIF]

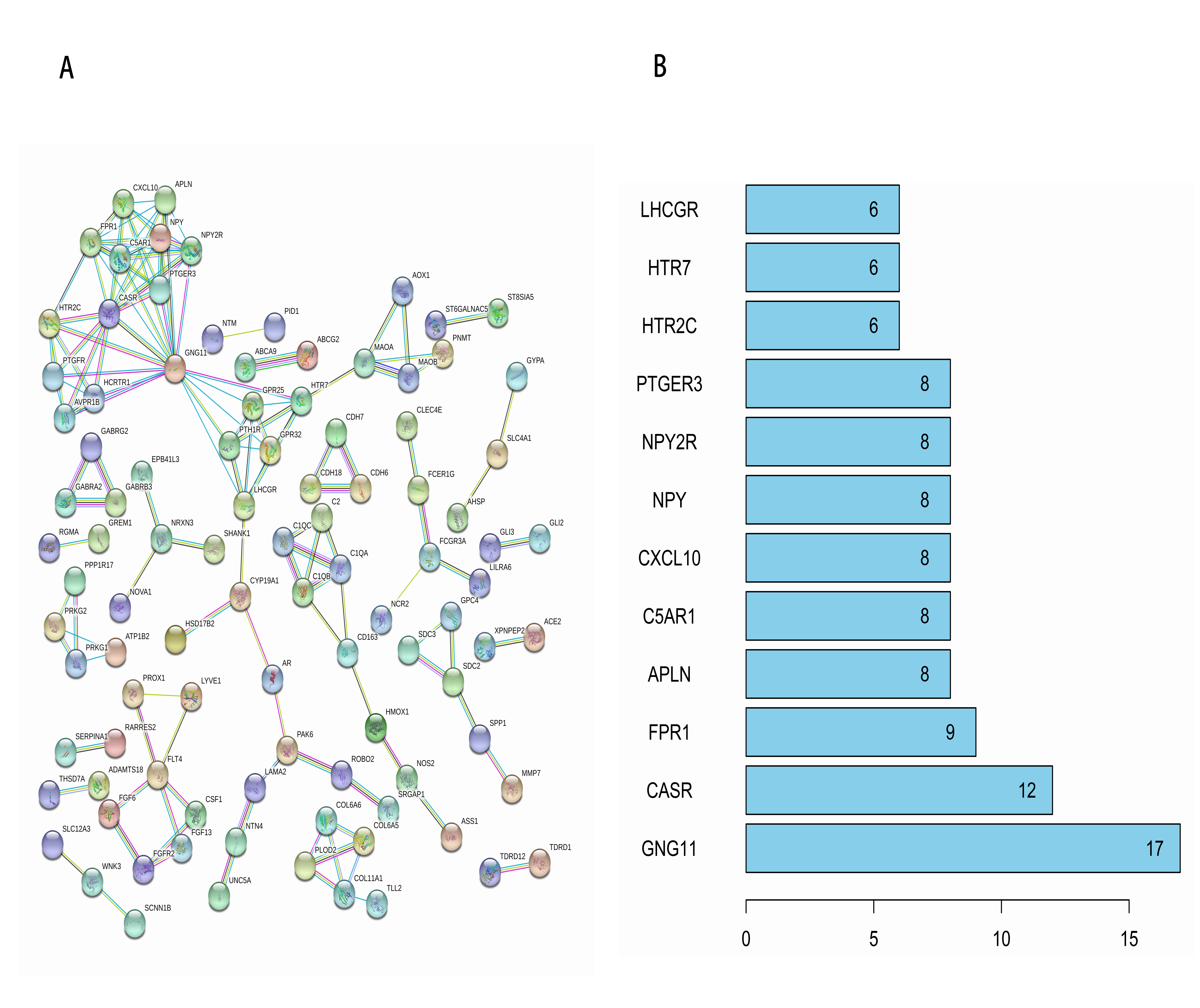

Supplement: Figure S12 — Protein–protein interaction network of differentially expressed intersection mRNAs associated with AQP1 methylation in elderly CN-AML patients (age > 60) (A) and 12 hub genes selected from protein–protein interaction network (B). The bar plot shows the enrichment scores of the interactions between the nodes. [file Image_12.TIF]

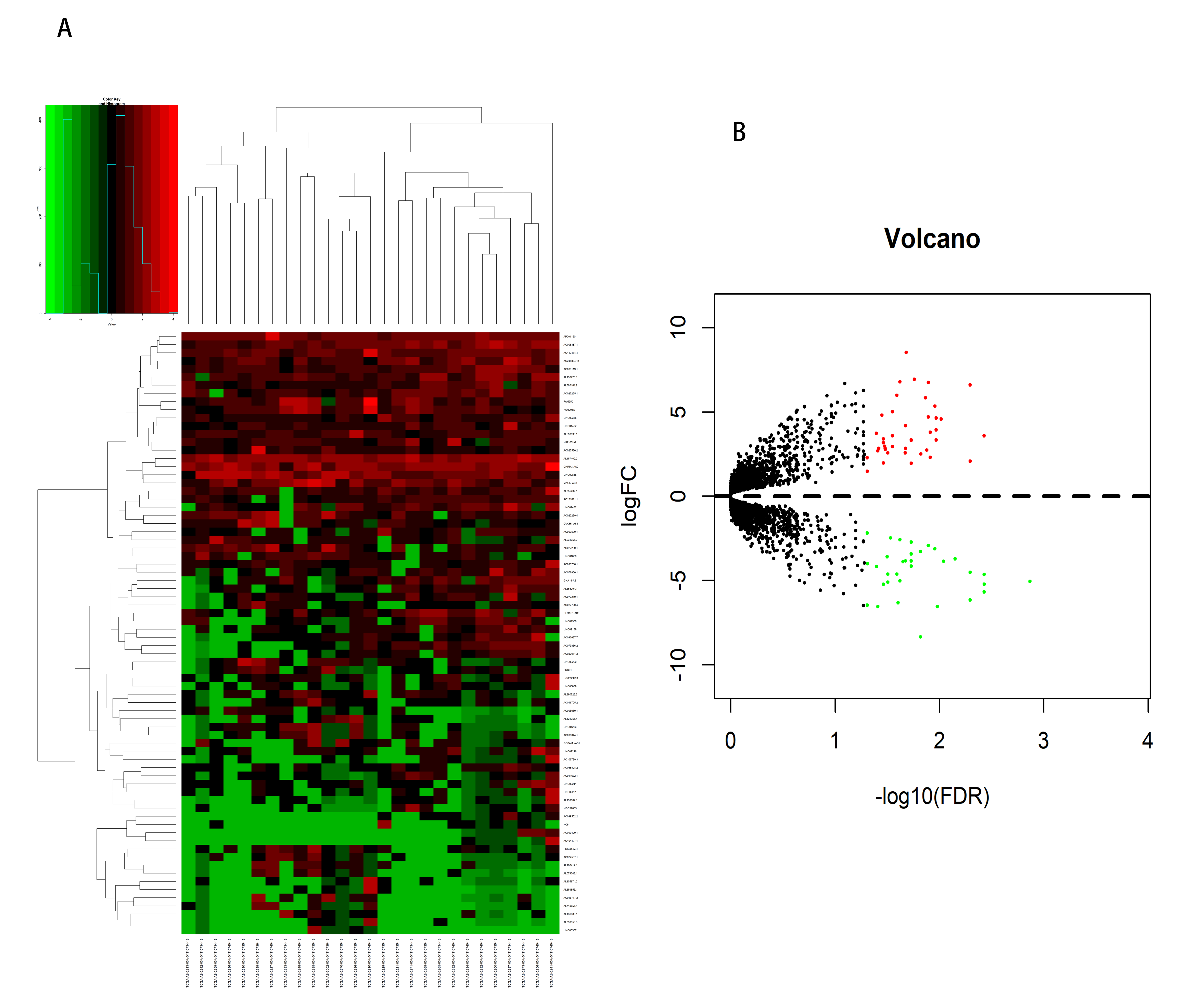

Supplement: Figure S13 — Heat map (A) and volcano plot (B) of the differentially expressed lncRNA between AQP1 hypermethylated and hypomethylated group. [file Image_13.TIF]

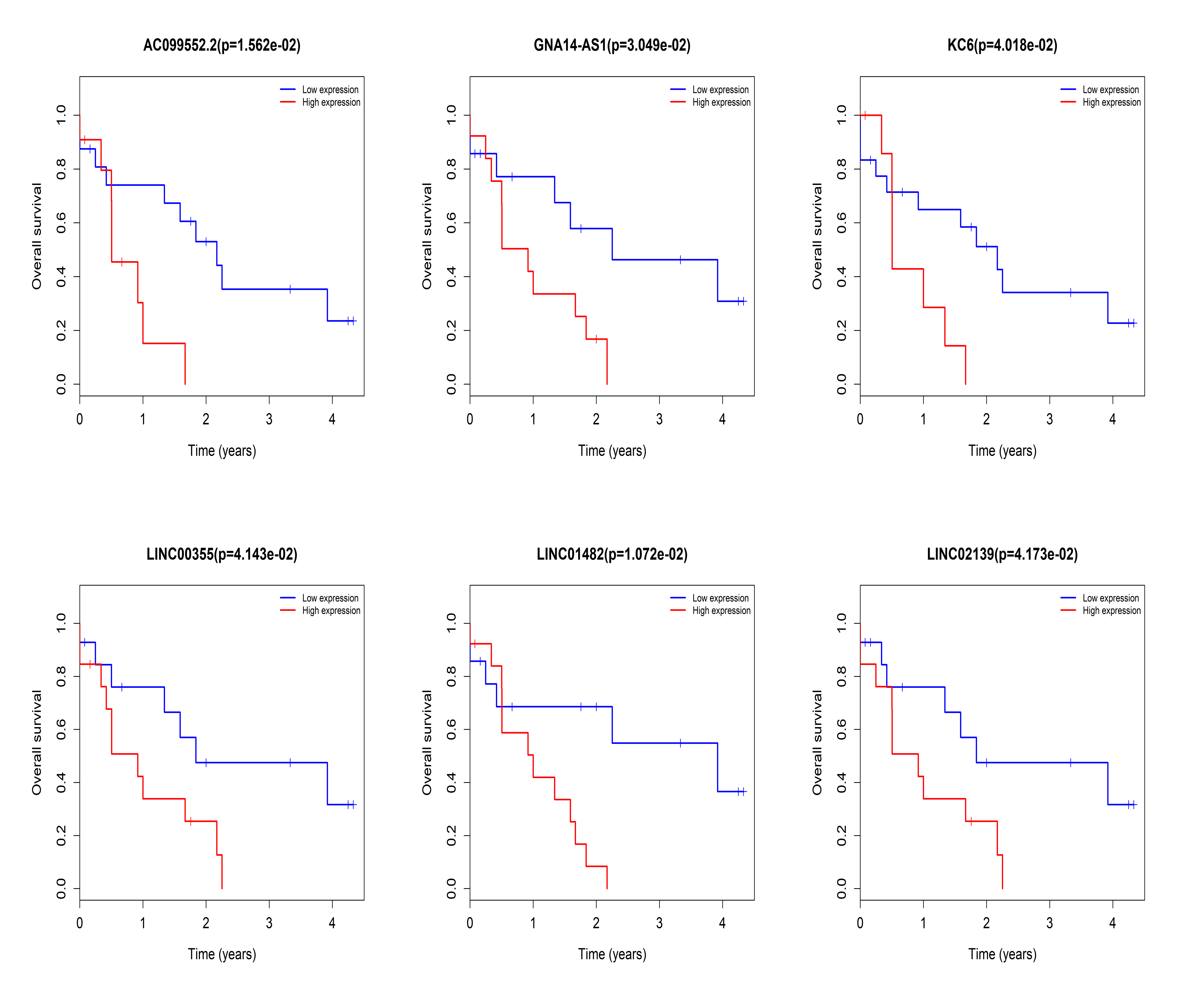

Supplement: Figure S14 — Kaplan-Meier survival curves for 6 lncRNAs associated with overall survival from the differentially expressed lncRNA between AQP1 hypermethylated and hypomethylated group. [file Image_14.TIF]

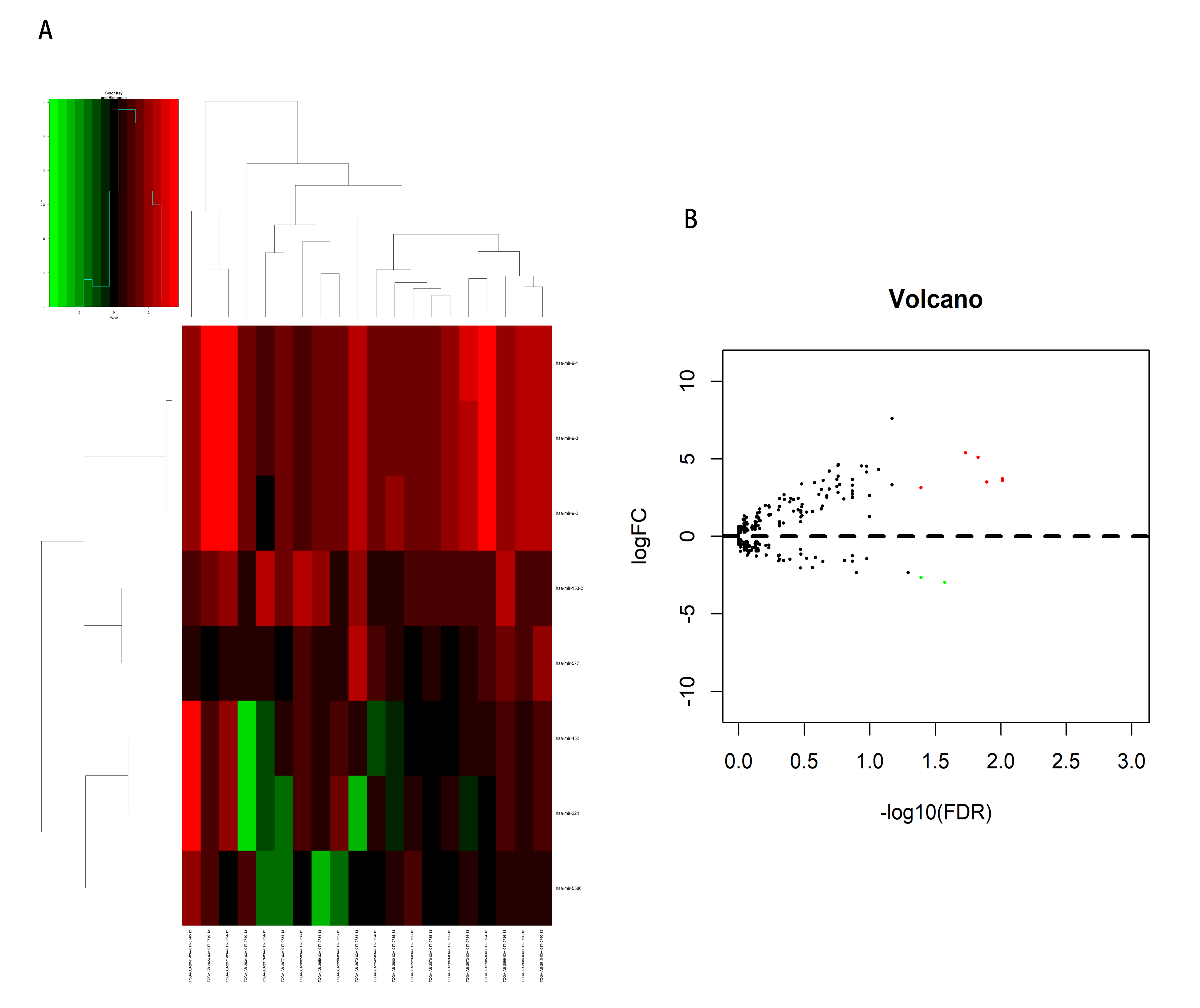

Supplement: Figure S15 — Heat map (A) and volcano plot (B) of the differentially expressed miRNA between AQP1 hypermethylated and hypomethylated group. [file Image_15.TIF]
