# Supplementary material for: A Novel Scoring System for Risk Assessment of Elderly Patients With Cytogenetically Normal Acute Myeloid Leukemia Based on Expression of Three AQP1 DNA Methylation-Associated Genes
Source: Front Oncol. 2020 Apr 21;10:566. doi: 10.3389/fonc.2020.00566 (PMC7186486; doi:10.3389/fonc.2020.00566)
Supplement: Table S1 — Univariate Cox analysis of clinical parameters with the prognosis in elderly CN-AML patients. [file Table_1.DOCX]

Table S1: Univariate Cox analysis of clinical parameters with the prognosis

| **Variants** | ***P*-value** |
| --- | --- |
| Age at diagnosis | 0.089 |
| Gender | 0.140 |
| Peripheral blasts | 0.204 |
| Bone marrow blasts | 0.996 |
| Platelets count | 0.745 |
| FAB classifications | 0.269 |
| FLT3 Mutation | 0.031 |
| IDH1 Mutation | 0.344 |
